# Safety netting in routine primary care consultations: an observational study using video-recorded UK consultations

**DOI:** 10.3399/bjgp19X706601

**Published:** 2019-11-19

**Authors:** Peter J Edwards, Matthew J Ridd, Emily Sanderson, Rebecca K Barnes

**Affiliations:** Centre for Academic Primary Care, Bristol Medical School, Population Health Sciences, University of Bristol, Bristol.; Centre for Academic Primary Care, Bristol Medical School, Population Health Sciences, University of Bristol, Bristol.; Centre for Academic Primary Care, Bristol Medical School, Population Health Sciences, University of Bristol, Bristol.; Centre for Academic Primary Care, Bristol Medical School, Population Health Sciences, University of Bristol, Bristol.

**Keywords:** health communication, patient safety, primary care, safety netting, video-recording

## Abstract

**Background:**

Safety-netting advice is information shared with a patient or their carer designed to help them identify the need to seek further medical help if their condition fails to improve, changes, or if they have concerns about their health.

**Aim:**

To assess when and how safety-netting advice is delivered in routine GP consultations.

**Design and setting:**

This was an observational study using 318 recorded GP consultations with adult patients in the UK.

**Method:**

A safety-netting coding tool was applied to all consultations. Logistic regression for the presence or absence of safety-netting advice was compared between patient, clinician, and problem variables.

**Results:**

A total of 390 episodes of safety-netting advice were observed in 205/318 (64.5%) consultations for 257/555 (46.3%) problems. Most advice was initiated by the GP (94.9%) and delivered in the treatment planning (52.1%) or closing (31.5%) consultation phases. Specific advice was delivered in almost half (47.2%) of episodes. Safety-netting advice was more likely to be present for problems that were acute (odds ratio [OR] 2.18, 95% confidence interval [CI] = 1.30 to 3.64), assessed first in the consultation (OR 2.94, 95% CI = 1.85 to 4.68) or assessed by GPs aged ≤49 years (OR 2.56, 95% CI = 1.45 to 4.51). Safety-netting advice was documented for only 109/242 (45.0%) problems.

**Conclusion:**

GPs appear to commonly give safety-netting advice, but the contingencies or actions required on the patient’s part may not always be specific or documented. The likelihood of safety-netting advice being delivered may vary according to characteristics of the problem or the GP. How to assess safety-netting outcomes in terms of patient benefits and harms does warrant further exploration.

## INTRODUCTION

Safety netting is a diagnostic strategy, utilised to manage clinical uncertainty, highlight ‘red flags’, and help monitor patients until their symptoms are explained.^1^^,^^2^ This broad term has been used to describe not only advice given during healthcare encounters, but also system and administration factors.^1^^,^^3^^,^^4^ This study assessed the communication of ‘safety-netting advice’, defined as: *‘Information shared with a patient or their carer designed to help them identify the need to seek further medical help if their condition fails to improve, changes, or if they have concerns about their health’*,^5^ which was adapted from Roland and colleagues’ definition.^6^

Recommendations to incorporate safety netting into everyday clinical practice are widespread.^3^^,^^7^ Safety netting is a key element of the Royal College of General Practitioners (RCGP) curriculum, features in multiple consultation models and clinical guidelines, and is recognised as forming part of ‘best practice’ in primary care.^3^^,^^8^^–^14 Conversely, a lack of safety netting has been implicated in contributing towards harm to patients, and GPs have been criticised for its omission.^15^^,^^16^

A consensus study indicates that clinicians agree safety netting should be employed in high-risk clinical situations, such as when the diagnosis is uncertain, the diagnosis carries a known risk of serious complications, or the individual patient has certain characteristics that puts them at an increased risk of illness or complications.^2^ Patients presenting to primary care may be regarded as having an inherently high rate of both risk and uncertainty as they often present early in the disease process, there is a low background prevalence of most diseases, and most GPs practise without immediate diagnostic investigations, such as X-rays and point-of-care blood tests. Neighbour first described a safety-netting checkpoint as one way of handling uncertainty.^12^ Recent research suggests safety netting is still valued by GPs when managing diagnostic uncertainty,^17^ but both doctors and patients have questioned the utility of generic or vague safety-netting advice.^2^^,^^18^

Many research studies on safety netting have relied on retrospective data collected in GP and patient interviews, survey data, and review of medical records.^1^^–^3^,^^19^^–^23 Qualitative research suggests that, despite a lack of training in safety-netting methods, GPs carry out safety netting ‘intuitively’ for acutely ill children.^19^^,^^20^ Yet the extent to which safety netting is utilised in everyday clinical practice in adult consultations, which type of problems warrant safety netting, and other factors that may contribute towards a GPs decision to safety-net or not are unclear.^24^

The aim of this explorative study was to describe when and how GPs deliver safety-netting advice in routine primary care consultations, the extent to which they document this advice in the medical notes, and to explore patient, GP, and problem factors associated with the presence or absence of safety-netting advice.

**Table:** How this fits in

Many studies of safety netting to date have relied on retrospective data collected in clinician and patient interviews, questionnaires, or review of medical records. Prior research has reported that GPs provide safety netting advice ‘intuitively’ in some circumstances, but it is not known exactly how and to what extent safety netting advice occurs in routine adult consultations. This is the first observational study to investigate when and how GPs give safety netting advice in routine consultations in the UK, and to identify factors associated with frequency of safety netting advice. This study confirms findings from prior qualitative research that safety netting advice is often not specific and not documented in the medical notes.

## METHOD

### Participants and data

This study was a secondary analysis of an existing primary care consultations archive from the ‘One in a Million’ study. Full details of data collection have been reported elsewhere.^25^^,^^26^ In short, the archive contains recordings and verbatim transcripts of unselected adult primary care consultations in areas of high and low deprivation in the West of England collected between 2014 and 2015, with permissions in place for reuse. Linked data include patient and GP characteristics, pre- and post-visit questionnaires, and electronic medical records.

### Problems raised in the consultations

All problems, defined as the answer to the question, *‘What is wrong?’*, raised in the consultations had previously been coded using the International Classification of Primary Care (ICPC-2).^27^ One coder, the first author, re-checked all the ICPC-2^28^ problem types for each consultation used in this project. Problems were coded under their diagnostic category where available, for example, a patient presenting with undifferentiated chest pain that was diagnosed to be musculoskeletal in origin would be coded under the ICPC-2 category of ‘Musculoskeletal L’. Where the diagnosis was ‘A97 No disease’ or ‘A85 Adverse effect medical agent’ then the problems were coded by the category of their presenting complaint. Problems were ordered chronologically according to the GP assessment of each problem.

### Screening and application of coding tool

Full details of the development and inter-rater reliability (IRR) of the coding tool are described elsewhere.^5^ Briefly, percentage agreement and Cohen’s κ scores for the presence or absence of safety-netting advice per consultation and per problem were 100% (κ = 1.0) and 89% (κ = 0.77) respectively. The mean agreement score for the application of the tool was 88% (κ = 0.66).^5^

One coder, the first author, screened all the consultations in the archive and counted the number of times safety-netting advice was delivered as well as which problems the advice applied to. Every consultation recording was viewed at least twice alongside the verbatim transcript. All problem codes and verbatim safety-netting advice were entered into the coding tool for full analysis. Additional codes capturing the wider context, for example, the presence or absence of follow-up, were assessed in all problems and not just those where safety-netting advice was present. Safety-netting advice was considered as contingent in nature and therefore distinguished from follow-up, which was defined as an unconditional future review, referral, or investigation of a problem.^5^

### Software and statistical analysis

Coding was undertaken using a Microsoft Excel spreadsheet that was imported into Stata (version 15.1) for data cleaning and statistical analysis. Descriptive statistics for when and how safety netting was delivered by consultation, problem, and for each discrete episode of safety-netting advice were calculated. Logistic regression was used to generate odds ratios (OR) for the frequency of safety-netting advice associated with different patient, clinician, or problem variants in both an unadjusted and adjusted model. In the adjusted model, multilevel mixed-effects modelling was used to adjust for all variables as covariates and for clusters within GP and patient. This adjusts for associations between variables, for example, if acute problems are more likely to be assessed first by the GP, all problems seen by the same GP, and multiple problems raised by the same patient. A significance level of 0.05 was used and 95% confidence intervals (CI) were calculated. Unless stated otherwise, OR are reported from the adjusted model. Patient problems with missing data were excluded from the adjusted models. All ORs reported for Index of Multiple Deprivation (IMD) quintiles used the least deprived quintile as the reference group.

## RESULTS

### Participants’ characteristics

The demographic information for the 318 patients who were included in this study are presented in Table 1. Almost two-thirds (63.5%, *n* = 202) were female and most were of a white ethnic group (87.1%, *n* = 277). In just under half of the consultations (47.5%) GPs assessed more than one problem. Of the 318 consultations 300 were video-recorded, 17 were audio only, and in one consultation the research team only had permission to use the transcript. Consultations were recorded with 23 GPs (13 female, 10 male, all white ethnic group) working in 12 practices.

**Table 1.** Patient characteristics, *N* = 318

**Characteristics**	***n* (%)**
**Sex**	
Male	116 (36.5)
Female	202 (63.5)

**Age, years**	
18–34	86 (27.0)
35–49	56 (17.6)
50–64	78 (24.6)
≥65	85 (26.7)
Not reported	13 (4.1)

**Ethnic group**	
White	277 (87.1)
Other	33 (10.4)
Not reported	8 (2.5)

**Problems per patient**	
1	167 (52.5)
2	89 (28.0)
3	47 (14.8)
≥4	15 (4.7)

**IMD quintile**	
1 (least deprived)	101 (31.8)
2	52 (16.3)
3	34 (10.7)
4	50 (15.7)
5 (most deprived)	80 (25.2)
Not reported	1 (0.3)

IMD = Index of Multiple Deprivation

### Safety-netting advice and follow-up frequencies

Safety-netting advice was present in 205/318 (64.5%) consultations but only 257/555 (46.3%) problems. However, most problems (468/555, 84.3%) had either some form of safety-netting advice or follow-up. For the 298 problems where no safety-netting advice was present, there was evidence of planned follow-up in 211 (70.8%) cases. The different types of planned follow-up are available (Supplementary Tables 1 and 2). Safety-netting advice varied by type of problem discussed (Table 2), being most common for neurological problems (16/27, 59.3%) and least common for urological disorders (4/21, 19.0%). On an individual GP basis (*n* = 23), safety-netting advice per problem assessed ranged from a minimum of 18.2% to a maximum of 89.5% with a mean average of 46.9% (standard deviation [SD] = 16.7%).

**Table 2.** Safety-netting and follow-up frequency by types of problem raised, *N* = 555

**Problem type (ICPC-2 code)**	**Problems,*n***	**Safety-netting advice present, %**	**Follow-up present, %**	**Safety-netting advice and/or follow-up present, %**
Neurological (N)	27	59.3	77.8	88.9
Digestive (D)	61	57.4	57.4	77.0
Ear (H)	14	57.1	42.9	85.7
Skin (S)	51	52.9	51.0	78.4
Cardiovascular (K)	46	52.2	67.4	84.8
Musculoskeletal (L)	96	50.0	69.8	87.5
Female genital (X)	25	48.0	68.0	92.0
Respiratory (R)	51	45.1	49.0	78.4
Blood, blood forming organs and immune mechanism (B)	9	44.4	88.9	100
Male genital (Y)	9	44.4	55.6	77.8
Eye (F)	7	42.9	71.4	100
Psychological (P)	67	40.3	88.1	92.5
Pregnancy, childbearing, family planning (W)	16	37.5	50.0	62.5
Endocrine/metabolic and nutritional (T)	32	31.3	81.3	84.4
General and unspecified (A)/process codes (-)	23	26.1	65.2	73.9
Urological (U)	21	19.0	90.5	95.2
**Total**	**555**	**46.3**	**67.2**	**84.3**

ICPC-2 = International Classification of Primary Care.^28^

### Safety-netting contextual codes

Diagnostic uncertainty was communicated for 256/555 (46.1%) of problems discussed, whereas the expected time course of the problem was communicated in only 127/555 (22.9%) of cases (Supplementary Table 1). The authors also recorded if the doctor issued any other contingency plans that did not meet this study’s definition of safety-netting advice such as contingent self-care, for example, *‘If the rash comes back just use this cream again’* (Supplementary Table 1).

### Content of safety-netting advice

There were 390 episodes of safety-netting advice observed across all consultations (Table 3). Most episodes were initiated by the GP (94.9%, *n* = 370) and delivered in the treatment planning (52.1%, *n* = 203) and closing phases of the consultation (31.5%, *n* = 123). Over half (52.8%, *n* = 206) of episodes were classified as generic, but notable; during treatment planning, there was a higher percentage of specific (114/203) rather than generic advice (89/203), whereas the advice delivered in closing was more commonly generic (90/123) rather than specific (33/123).

**Table 3.** Content of safety-netting advice across all episodes, *N* = 390

**Content**	**Codes^a^**	**Episode frequency,*n* (%)**
**Applicable to problem,treatment or management, or both**	Problem	270 (69.2)
	Treatment or management	38 (9.7)
	Both	82 (21.0)

**Stage of consultation**	Establishing reason for consultation	1 (0.3)
	Gathering information	24 (6.2)
	Delivering diagnosis	36 (9.2)
	Treatment planning	203 (52.1)
	Closing	123 (31.5)
	Unclear	3 (0.8)

**Initiation**	Patient	20 (5.1)
	GP	370 (94.9)

**Format**	Conditional plus course of action	378 (96.9)
	Conditional warning only	12 (3.1)

**Strength of endorsement**	Weaker (can, could)	67 (17.2)
	Neutral	262 (67.2)
	Stronger, for example, must, should	61 (15.6)

**Number of conditionals or symptomsto look out for, for example,worsening pain, symptoms persist,ornew weakness**	Implicit conditional^b^	5 (1.3)
	1	234 (60.0)
	2	77 (19.7)
	3	36 (9.2)
	4	18 (4.6)
	≥5	20 (5.1)

**Generic or specific advice**	Specific (cough up blood, chest pain …)	184 (47.2)
	Generic (problems, issues, concerns, worse)	206 (52.8)

**Action advised**	No action (conditional warning only)	12 (3.1)
	Contact other in-hours medical service	12 (3.1)
	Return to practice	244 (62.6)
	Return to same GP	109 (27.9)
	Contact OOH service	6 (1.5)
	Contact emergency services	7 (1.8)

**Focus of action**	No action (conditional warning only)	12 (3.1)
	Patient (*‘****you*** *come back ’*)	163 (41.8)
	GP (*‘****I*** *will have another look at it’*)	146 (37.4)
	Both (*‘****you*** *come back, and* ***I*** *will have another look at it’*)	69 (17.7)

**Timescale of action**	Not specified	303 (77.7)
	Named/fixed time (*‘2 weeks’*)	65 (16.7)
	Immediate (*‘go straight to A&E’*)	22 (5.6)

**Patient response at the endof the safety-netting advice**	No response^c^	40 (10.3)
	Resists/misaligns	37 (9.5)
	Nods only	42 (10.8)
	Acknowledgement/acceptance	271 (69.5)

a See the codebook for further explanation and examples of all codes. The safety-netting advice codebook prepared by the authors is available as Supplementary Table 3.

b Example: ‘So 3 months if not before’.

c Six cases of no response in audio only so unable to determine if the patient was nodding. A&E = accident and emergency department. OOH = out of hours.

In most cases, GPs advised patients to return back to their primary care team (90.5%; return to practice and return to same GP data [244 + 109]/390) but a timescale of when to seek medical help was not often specified (77.7%, *n* = 303). Rarely, a fixed period was given (16.7%, *n* = 65):

GP: ‘If the symptoms are persisting and you are no better you do need to come back and see me, I’ll say 2 weeks.’

or the patients were informed to take immediate action (5.6%, *n* = 22):

GP: ‘If you’re sitting there thinking, “I’m really bad”, don’t think, “I’ll wait till tomorrow”. I am telling you now you need to call somebody straight away.’

After the safety-netting advice had been delivered patients most commonly responded with a simple acknowledgement, for example, ‘*Mmhm* ’, ‘*Yeah*’, or clear acceptance, for example, *‘OK’* (69.5%, *n* = 271). However, in 9.5% (*n* = 37) there were signs of resistance or misalignment, where patients chose to reject the advice or questioned the GP further:

GP: ‘Any problems, then you know where we are.’

Patient: ‘Don’t say things like that.’

It was equally rare (9.6%, 23/240 problems: 17 problems with generic safety-netting advice applying to multiple problems not included to avoid double counting) that patients asked any questions about the safety-netting advice.

### Mode of communication

Safety-netting advice was most commonly only communicated verbally (249/257 problems, 96.9%). Eight problems were identified as having both verbal and written safety-netting advice. There were nine problems where GPs gave patients a written information leaflet that may have contained safety-netting advice, but the authors were unable to ascertain the exact contents of the leaflet and the GP did not vocalise that the leaflet contained safety-netting advice.

### Documentation

Where safety-netting advice was given for a problem and medical records were available (242/257, 94.2%), there was evidence in the medical notes that the patient had been given safety-netting advice in only 109/242 (45.0%) of cases. Documentation rates of any follow-up plans verbalised in the consultation for each problem and where medical records were available were higher at 295/354 (83.3%).

### Symptoms or conditions

The most common conditions or symptoms, for example, ‘*if x happens then …’*, highlighted in the safety-netting advice for all problems are listed in Table 4. The most common verbalised category was a new specific symptom or condition (197/692), which applied to 87 problems, indicating that doctors often listed multiple symptoms for patients to look out for when assessing one problem. The most common category per problem was if the current illness or symptoms persisted. There were 179 verbalised conditions in the persisting category, which applied to 106 problems indicating that doctors often repeated the need for the patient to seek help if their symptoms persisted for the same problem. There were 49 incidents where the doctors vocalised if the patient had any ‘problems’ or ‘issues’ to seek medical help. This applied to 55 medical problems, as its generic nature covers multiple types of problems assessed in the same consultation. The mean number of symptoms or conditions per discrete safety-netting advice episode was 1.77 (692/390) with a range of 1–10 (SD = 1.33).

**Table 4.** Safety-netting advice conditions/symptoms to look out for

**Category**	**Frequency verbalised in all consultations *n***	**Frequency per problem (*N*= 555)*n* (%)**
**New specific symptom or condition**		
*‘Skin starts to break down ’, ‘cough up any blood ’, ‘indigestion pains’*	197	87 (15.7)

**Current illness or symptoms persist**		
*‘If you feel it ’s no better in a fortnight, come back and see me.’*	179	106 (19.1)

**Current illness or symptoms worsen**		
*‘If you feel by all means that things have got worse* […] *let us know and we’ll see her sooner.’*	74	50 (9.0)

**Other non-specific condition**		
Develop new *‘symptoms’, ‘want to come back’, ‘not tolerating it’,* *‘getting fed up’, ‘questions’*	64	54 (9.7)

**Any ‘problems’/‘issues’**		
*‘Any problems in the meanwhile, give me a shout.’*	49	55 (9.9)

**Return of previous symptoms**		
*‘Come back, please, if you have any return of your symptoms.’*	41	27 (4.9)

**Need**		
*‘I’ll see you in 2 months or sooner if need be.’*	23	23 (4.1)

**Concerned, worried, or struggling**		
*‘If you’re worried, about any of that, come back to me.’*	18	18 (3.2)

**Current condition changes**		
*‘If anything has changed in the interim, we’ll see you again.’*	17	14 (2.5)

**Change in ‘wellness’**		
*‘If you’re feeling unwell, then leave me a message and I’ll ring you back.’*	9	8 (1.4)

**Have not heard about a referral/appointment**		
*‘You should hear within the next couple of weeks. If you haven’t heard* *anything, you can let us know and we can chase that up for you.’* (2-week wait skin cancer referral)	9	7 (1.3)

**Starts to limit function**		
*‘If it becomes* […] *so painful you can’t walk, come back.’*	7	7 (1.3)

**Implicit conditional**		
*‘So, 3 months, if not before.’*	5	9 (1.6)

**All symptoms/conditions**	692	257 (46.3)

### Patient, GP, and problem factors associated with safety-netting advice

Acute problems, including ‘acute on chronic’ problems, for example, acute shortness of breath attributed to chronic obstructive pulmonary disease (COPD), were more likely to be given safety-netting advice than chronic problems; for example, a general review of COPD (OR 2.18, 95% CI = 1.30 to 3.64, *P* = 0.003), in both the adjusted and unadjusted model (Table 5). Problems assessed by the GP first were more likely to be given safety-netting advice compared with problems assessed later in the consultation (OR 2.94, 95% CI = 1.85 to 4.68, *P*<0.001). To ensure this association was not driven purely by consultations where only one problem was discussed, the analysis was repeated including only problems from consultations where multiple problems were assessed and still found strong evidence of an association (OR 2.40, *P* = 0.001). There was weak evidence of some form of follow-up being associated with less safety-netting advice (OR 0.63, 95% CI = 0.38 to 1.02, *P* = 0.059) (Table 5).

**Table 5.** Problem, patient, and GP variants as predictors of safety-netting advice

**Codes from observingconsultation/linked data**	**Options**	**Safety-netting advice present*n*/*N* (%)**	**Unadjusted model OR^a^ (95% CI)**	***P*-value**	**Adjusted model OR^a^ (95% CI)**	***P*-value^a^**
**Is this problem acute, acute on chronic, or chronic?**	Acute/AoC	182/342 (53.2)	2.09 (1.47 to 2.98)	<0.001	2.18 (1.30 to 3.64)	0.003
	Chronic	75/213 (35.2)				

**Is this the first presentation with this medical problem to a healthcare professional?**	First presentation	61/114 (53.5)	1.40 (0.93 to 2.12)	0.111	1.08 (0.61 to 1.91)	0.800
	Not first presentation	188/417 (45.1)				
	Unclear (excluded)	8/24 (33.3)				

**Is the problem the first assessed in the consultation or after the first?**	First/only	181/318 (56.9)	2.80 (1.97 to 3.98)	<0.001	2.94 (1.85 to 4.68)	<0.001
	After the first	76/237 (32.1)				

**Is there evidence for any follow-up for this problem?**	Follow-up present	162/373 (43.4)	0.70 (0.49 to 1.00)	0.052	0.63 (0.38 to 1.02)	0.059
	No evidence of follow-up	95/182 (52.2)				

**What was the age of the doctor who assessed this problem?**	Aged ≤49 years	185/354 (52.3)	1.96 (1.37 to 2.80)	<0.001	2.56 (1.45 to 4.51)	0.001
	Aged ≥50 years	72/201 (35.8)				

**Is this problem assessed in an older patient?**	Aged ≥65 years	65/148 (43.9)	0.96 (0.66 to 1.41)	0.851	1.21 (0.69 to 2.12)	0.503
	Aged 18–64 years	173/386 (44.8)				
	Unreported (excluded)	19/21 (90.5)				

**What is the sex of the patient presenting with this problem?**	Female	157/343 (45.8)	0.95 (0.67 to 1.33)	0.748	0.83 (0.50 to 1.36)	0.458
	Male	100/212 (47.2)				

**What is the ethnicity of the patient presenting with this problem?**	Other	34/57 (59.6)	1.88 (1.08 to 3.29)	0.027	1.44 (0.60 to 3.44)	0.412
	White	213/484 (44.0)				
	Unreported (excluded)	10/14 (71.4)				

**What is the IMD quintile of the patient presenting with this problem?**	1 (least deprived)	87/159 (54.7)	1.00 (Ref)		1.00 (Ref)	
	2	38/88 (43.2)	0.63 (0.37 to 1.06)	0.083	0.75 (0.35 to 1.58)	0.444
	3	28/53 (52.8)	0.93 (0.50 to 1.73)	0.811	0.97 (0.40 to 2.38)	0.953
	4	37/94 (39.4)	0.54 (0.32 to 0.90)	0.019	0.59 (0.28 to 1.24)	0.162
	5 (most deprived)	66/160 (41.3)	0.58 (0.37 to 0.91)	0.016	0.90 (0.44 to 1.84)	0.771
	Unreported (excluded)	1/1 (100)	—	—	—	—

a Odd ratios and 95% CIs generated from logistic regression of variants associated with the presence or absence of safety-netting advice for problems raised in the archive. Multilevel modelling adjusts for all variables in table and for within clustering by GP and patient (all problems seen by same GP and problems from the same patient). Adjusted P-values exclude problems with unreported data, n = 505 problems. AoC = acute on chronic. CI = confidence interval. IMD = Index of Multiple Deprivation. OR = odds ratio. Ref = reference.

Frequency of safety-netting advice was not statistically significantly higher for problems presented by older patients (aged ≥65 years, OR 1.21, 95% CI = 0.69 to 2.12, *P* = 0.503; aged ≥75 years, OR 1.29, *P* = 0.518). In the unadjusted models it appeared as if problems raised by patients who were not of a white ethnicity (OR 1.88, 95% CI = 1.08 to 3.29, *P* = 0.027) or those raised by patients from the most deprived IMD quintile (OR 0.58, *P* = 0.016 were associated with an altered frequency of safety-netting advice; however, these associations were not maintained in the adjusted model (OR 1.44, 95% CI = 0.60 to 3.44, *P* = 0.412 and OR 0.90, CI = 0.44 to 1.84, *P* = 0.771 respectively) (Table 5).

As logistic regression for the presence or absence of safety-netting advice using GPs’ age as a continuous variable showed an association for younger GPs to have increased odds of giving safety-netting advice, the GPs were categorised into two groups, aged ≥50 years or aged ≤49 years. Problems assessed by GPs ≤49 years of age were more likely to have safety-netting advice (OR 2.56, 95% CI = 1.45 to 4.51, *P* = 0.001) compared with problems assessed by GPs aged ≥50 years.

## DISCUSSION

### Summary

Safety-netting advice was present in just under two-thirds of consultations but applied to just under half of all problems assessed during these consultations. Acute problems, problems assessed first by the GP, and problems assessed by GPs aged ≤49 years were more likely to be issued safety-netting advice. Most safety-netting advice was initiated by the GP. Specific advice was commonly delivered during the treatment planning phase whereas generic advice tended to be delivered during the closing phase. The most common eventuality patients were told to look out for per problem was if their current symptoms persisted. Patients were rarely given written advice, and, when safety-netting advice had been given, for just over half of problems there was no documentation in the medical notes.

### Strengths and limitations

To the authors’ knowledge, this is the first observational study to assess when and how GPs give safety-netting advice in routine consultations with adult patients in the UK; and to assess what type of problem, patient, and GP factors are associated with safety-netting advice. Each consultation was viewed at least twice alongside a verbatim transcript to ensure coding accuracy, and systematic methods were utilised to check for missing codes. The authors utilised a coding tool that was specifically designed to assess safety-netting advice in primary care, generated from the published literature and systematic observations of real-life consultations to generate codes with substantial levels of IRR.^5^ However, apart from the IRR testing, all coding was completed by only one coder.

Although the act of recording itself may change the communication between participants,^29^ a review by Themessl-Huber and colleagues concluded that there was little evidence that audio- or video-recording significantly affects practitioner or patient behaviour.^30^ Indeed, patients often forget during the consultation that they are being recorded.^31^ Furthermore, this is a secondary analysis of a dataset and, though the participants were aware their consultations may be used in future research projects, they were not specifically aware that how they gave safety-netting advice was going to be evaluated, making it more likely the present results represent the true day-to-day practice of individuals involved in the ‘One in a Million’ study.

This current study involved 23 GPs in one region of England recorded between 2014 and 2015, so the results are unlikely to be generalisable to all GPs in the UK, who may be working with different patient populations and under very different circumstances. There was a lack of ethnic diversity in this study’s dataset with all GPs and most patients (87%) reporting being of a white ethnicity. The effects of variations in training, cultural, and social norms are likely to influence safety-netting behaviours in different geographical areas and warrant further exploration.

Furthermore, when generic advice by the GP was given at the end of the consultation where multiple problems were discussed, for example, *‘any problems let me know’*, then all problems within the consultation were coded as having been given safety-netting advice. Giving the benefit of the doubt here may have overestimated the prevalence of safety-netting advice but in most cases it not possible to ascertain if the doctor was referring to the final problem that was discussed or all problems during the consultation.

This study identified that chronic problems were less likely to be given safety-netting advice. However, a limitation of the dataset is that it is not possible to tell if the patient had previously been given safety-netting advice in another consultation for the same problem. Likewise, follow-up plans for chronic problems may have already been arranged that were not discussed in the recorded consultation and in some conditions patients are automatically invited to attend for an annual review, such as asthma and COPD, therefore, the present study may have underestimated the amount of follow-up for each problem.

In this study robust statistical analysis was used to adjust for covariants and clustering when exploring GP, patient, and problem factors associated with the frequency of safety-netting advice. The importance of adjusting is demonstrated by the unadjusted association between non-white ethnicity and higher frequency of safety-netting advice (*P* = 0.027), evidence for which becomes very weak (*P* = 0.412) in the adjusted model.

### Comparison with existing literature

Overall, rates of safety-netting advice in this study were comparable with one other primary care study that reported on ‘safety-netting’ while assessing the extent of patient recall of the content of face-to-face and telephone consultations.^32^

There was weak evidence (*P* = 0.059) that, where some form of follow-up was discussed, problems had less safety-netting advice (Table 5). This may indicate that some doctors are not discriminating between safety-netting advice and follow-up planning, and recent research has suggested classifying them on the same spectrum.^1^^,^^3^ However, there were 162/555 problems (29.2%) (Supplementary Table 2) that had both safety-netting advice and follow-up, indicating that GPs in the present study recognised the need for both ‘conditioned follow-ups’,^33^ referred to in this article as safety-netting advice, and unconditional review or investigation of patients (planned follow-up).

Consultation models usually indicate that safety netting should be delivered towards the end of the consultation, which was consistent with the present results and one other study in Danish primary care.^12^^,^^13^^,^^33^ Similarly, the finding that generic advice is more commonly given when closing the consultation is consistent with anecdotal evidence from clinicians.^2^ Currently, the benefits of generic advice, for example, *‘any problems let me know’*, when patients already have the right to contact their GP about any issues are unknown and require further evaluation. However, parents of sick children have reported that they often consider safety-netting advice too vague to be useful.^18^

The low rates of documentation of safety netting in the medical record observed in this study are consistent with a previous qualitative study report.^20^ Consequently, other research studies, such as audits of safety-netting practices from medical records, may underestimate the true incidence of safety netting in primary care.^34^

### Implications for research and practice

Intervention studies comparing enhanced safety-netting communication practices with usual care may be the best route to evaluate effectiveness. A more in-depth analysis of safety-netting communication practices and patient responses would provide further evidence for the design of such communication-based interventions and for intervention training. Any such studies would preferably be set in a more controlled context where it would be expected to see safety-netting activity, such as those with low risk but not no risk of cancer.^35^

It is unclear from the present study if outcomes differed between patients who were given safety-netting advice and those who were not. Future observational studies may benefit from longer-term follow-up of patients presenting with a less diverse array of medical problems to be powered to evaluate whether safety-netting advice alters patient outcomes.

Even within the small sample of 23 GPs in this study, there was wide variation in clinical practice (rates of safety-netting advice per problem ranged between 18.2% and 89.5%). This may raise questions about doctors’ training in safety-netting methods. However, if GPs are going to be held to clinical standards that they ‘should’ give safety-netting advice for certain conditions, further guidance is required on exactly what type, when, and how advice and information ought to be shared with patients. Furthermore, the low documentation rates indicate that GPs may be putting themselves at unwarranted medicolegal risk. Automated documentation systems may help alleviate some of this risk in a time-pressured environment. Increasing the ease of access to written patient information leaflets that include specific safety-netting advice may also help to increase both the amount of written and specific advice issued to patients. Finally, a consensus among clinicians, researchers, and patients of what exactly constitutes effective safety netting is required alongside robust evaluation if safety netting is to be considered part of evidence-based medicine and an accountable standard.
